# DEPRESSION AND HYPERTENSION AS RISK FACTORS OF CARDIOVASCULAR EVENTS AMONG MIDDLE-AGED AND OLDER ADULTS IN CHINA

**DOI:** 10.1093/geroni/igz038.1894

**Published:** 2019-11-08

**Authors:** Lydia Li, Zhenmei Zhang, Hongwei Xu, Jinyu Liu

**Affiliations:** 1 University of Michigan, Ann Arbor, Michigan, United States; 2 Michigan State University, Michigan, United States; 3 Queens College in the City University of New York, New York, United States; 4 Columbia University, New York City, New York, United States

## Abstract

Objectives: 1) Examine effects of depression and hypertension on cardiovascular events (CV) in a two-year period. 2) Explore urban and rural differences Methods: Data from the first two waves of Chinese Health and Retirement Longitudinal Study, with a national sample of 14,560 adults age 45+, were used. The dependent variable is whether a CV (defined as heart attack or stroke) occurred between baseline and W2 (1=Yes, 0=No), based on respondents’ report at W2. Depression was dichotomized using a score of 12 on the 10-item CES-D. Hypertension was based on self-report. Logistic regression was conducted. Covariates included sociodemographic characteristics and nine other chronic conditions. All independent variables were measured at baseline. Results: About 5.3% (n=768) of the sample had a CV between baseline and W2. Depression increases the risks of CV by 67% for rural (OR=1.67, 95% CI=1.3, 2.12) and 42% for urban respondents (OR=1.42, 95% CI=1.05, 1.91). Hypertension increases the risk by 51% for rural (OR=1.51, 95%CI=1.18, 1.94) but is not statistically significant among urban respondents. Interaction effects of hypertension and residential areas are statistically significant (χ2 (1) = 6.44, p = .01) Conclusion and Discussion: Given the high cost associated with heart attack and stroke, treating depression is an effective approach to reduce health care cost. Hypertension increases the risk of CV for rural but not urban respondents. It may be that hypertension is not as well managed among rural residents as in their urban counterparts. Improving hypertension management among rural residents should be a priority in China.

